# New insights and best practices for the successful use of Empirical Mode Decomposition, Iterative Filtering and derived algorithms

**DOI:** 10.1038/s41598-020-72193-2

**Published:** 2020-09-16

**Authors:** Angela Stallone, Antonio Cicone, Massimo Materassi

**Affiliations:** 1 Istituto Nazionale di Geofisica e Vulcanologia (INGV), Via di Vigna Murata 605, 00143 Roma, Italy; 2 Istituto di Astrofisica e Planetologia Spaziali dell’Istituto Nazionale di Astrofisica (IAPS-INAF), Via Fosso del Cavaliere 100, 00133 Roma, Italy; 3 Istituto dei Sistemi Complessi del Consiglio Nazionale delle Ricerche (ISC-CNR), Via Madonna del Piano 10, 50019 Sesto Fiorentino (Firenze), Italy

**Keywords:** Applied mathematics, Computational science, Atmospheric dynamics, Projection and prediction, Hydrology, Palaeoclimate, Hydrology, Atmospheric dynamics, Hydrology, Seismology, Volcanology, Geology, Geomagnetism, Geophysics, Hydrogeology, Palaeomagnetism, Sedimentology, Seismology, Volcanology, Astronomical instrumentation, Magnetospheric physics, Solar physics, Arrhythmias, Congenital heart defects, Heart failure, Arrhythmias, Congenital heart defects, Heart failure, Headache, Migraine, Parkinson's disease, Sleep disorders, Rheumatoid arthritis, Spondyloarthritis, Systemic lupus erythematosus, Astronomical instrumentation, Astrophysical magnetic fields, General relativity and gravity, Time-domain astronomy, Transient astrophysical phenomena, Atmospheric dynamics, Geodynamics, Hydrology, Seismology, Volcanology, Astronomical instrumentation, Magnetospheric physics, Solar physics, Energy grids and networks, Power distribution, Energy grids and networks, Power distribution, Aerospace engineering, Civil engineering, Electrical and electronic engineering, Mechanical engineering, Optical spectroscopy, Acoustics, Astronomical instrumentation, Astrophysical magnetic fields, General relativity and gravity, Time-domain astronomy, Transient astrophysical phenomena, Fluid dynamics, Information theory and computation, High-harmonic generation, Magneto-optics, Nonlinear optics, Astrophysical plasmas, Magnetically confined plasmas, Astronomical instrumentation, Magnetospheric physics, Solar physics, Nonlinear phenomena, Imaging techniques

## Abstract

Algorithms based on Empirical Mode Decomposition (EMD) and Iterative Filtering (IF) are largely implemented for representing a signal as superposition of simpler *well-behaved* components called Intrinsic Mode Functions (IMFs). Although they are more suitable than traditional methods for the analysis of nonlinear and nonstationary signals, they could be easily misused if their known limitations, together with the assumptions they rely on, are not carefully considered. In this work, we examine the main pitfalls and provide caveats for the proper use of the EMD- and IF-based algorithms. Specifically, we address the problems related to boundary errors, to the presence of spikes or jumps in the signal and to the decomposition of highly-stochastic signals. The consequences of an improper usage of these techniques are discussed and clarified also by analysing real data and performing numerical simulations. Finally, we provide the reader with the best practices to maximize the quality and meaningfulness of the decomposition produced by these techniques. In particular, a technique for the extension of signal to reduce the boundary effects is proposed; a careful handling of spikes and jumps in the signal is suggested; the concept of multi-scale statistical analysis is presented to treat highly stochastic signals.

## Introduction

Nonstationary processes and signals generated by nonlinear dynamics are ubiquitous in real life. Their time-frequency analysis and features extraction can help in solving open problems in many fields of research.

However, when dealing with nonlinear and nonstationary time series, neither the standard Fourier transform^1^ nor the wavelet Transform represent the best approach. In fact, all of them produce linear decompositions, whereas real life data sets are in many cases generated by nonlinear phenomena. Furthermore, all the aforementioned methods have troubles providing an accurate time-frequency representation of the data^2^ due to the well known Heisenberg uncertainty principle^3^. For all these reasons, several methods have been proposed to increase the accuracy of the time-frequency representation produced by the previously mentioned methods, like the Short Time Fourier Transform (STFT)^3^, the Synchrosqueezed Wavelet Transform^4,5^ or the ConceFT method^6^.

Two decades ago a different kind of method called Empirical Mode Decomposition (EMD) was introduced by Huang and his collaborators in the seminal work published in 1998^7^. This method is aimed at the decomposition of nonstationary and nonlinear signals in order to unravel their hidden quasi-periodicity and features. It is a local and adaptive data-driven method which makes it a much more suitable technique for nonlinear and nonstationary data analysis.

Furthermore, it has a *divide et impera* approach which allows to bypass the Heisenberg-Gabor uncertainty principle^8^. First, the signal is divided into several simple components via the so called sifting approach, which boils down to the calculation of the signal moving average via envelopes connecting its extrema. Then, each component is analysed separately in the time-frequency domain^9^.

While the EMD method proved to be extremely powerful in extracting simple components from a given signal, it is unstable to perturbations^10^ and susceptible to mode splitting and mode mixing^11^. These are the reasons why the Ensemble Empirical Mode Decomposition (EEMD) method^10^ first, and then several alternative noise-assisted EMD-based methods (e.g. the complementary EEMD^12^, the complete EEMD^13^, the partly EEMD^14^, the noise assisted multivariate EMD (NA-MEMD)^15^, and fast multivariate EMD (FMEMD)^16^) have been proposed.

While these newly developed methods are based on EMD, they all address the so called mode mixing problem and guarantee the stability of the decomposition with respect to noise^9^. However, mode splitting is still an open problem for all these methods and, more importantly, their mathematical analysis is by no means complete^9^.

An alternative technique for signal decomposition, based on iterations like the EMD, is the so called Iterative Filtering (IF) method, proposed by Lin et al. in 2009^17^. The IF algorithm structure is based on the EMD one, but it differs from it in the way it computes the signal moving average, which is derived as a point by point local weighted average. This is obtained convolving the signal with an a priori chosen “filter function”, which is simply any positive and compactly supported function whose area equals one. To guarantee a priori the convergence of this method it has been recently proved that it is sufficient to consider a filter function obtained as convolution of another filter function with itself^18^.

IF method allows to produce results similar to the EMD-based algorithms, but with the important advantage that it is possible to guarantee a priori its convergence and stability. This is due to the moving average computation based on convolution, which has opened the door to the mathematical analysis of IF and derived algorithms^18–22^. Furthermore, IF mathematical analysis has also led to its acceleration via Fast Fourier Transform (FFT) in what is called the Fast Iterative Filtering (FIF) method^18,23^.

We point out that IF and FIF methods do not suffer of mode mixing^24^, whereas mode splitting can be easily avoided by tuning the value of the stopping criterion parameter^2^.

The IF algorithm has been generalized to tackle highly nonstationary signals (leading to the Adaptive Local Iterative Filtering (ALIF)^25,26^), and, as for EMD, multidimensional signals^27,28^, and multivariate ones^29^.

Both the EMD- and IF-based algorithms yield a decomposition of any signal in simpler components, known as Intrinsic Mode Functions (IMFs) which fulfill two conditions: the envelopes connecting the minima and maxima of an IMF have a local average which equals zero; the IMF extrema number differs from the number of its zero crossing of at most one. Differently from the sine and cosine components of the Fourier transform, the IMFs are oscillatory modes whose amplitude and frequency can vary over time. Their instantaneous frequency estimation done, for instance, via Hilbert transform^30^, provides an accurate time-frequency representation of a nonstationary and nonlinear signal.

The versatility of these techniques has opened the door to their application in many applied fields. As a matter of fact, they are largely implemented in geophysical studies^30–32^, with applications in Seismology (data denoising and/or detrending^33–35^, pre-seismic signal analysis^36–38^, earthquake-induced co/post-seismic anomalies analysis^39^), Exploration Seismology (for improving signal-to-noise ratio in seismic data processing routines^40,41^ or for seismic interpretation^42^), Geomagnetism^43–49^, Engineering Seismology (mainly for analysing ground motion data^50–54^), climate, atmospheric and oceanographic sciences^55–60^. Their use is also common in Physics (for data analysis^61–65^, data denoising and/or detrending^66–68^, to assess causal relationships between two time series^69^, or to extract information on multiple time scales^70^); Medicine and Biology^71–79^; Engineering^80–85^; Economics and Finance^86–88^; Computer vision^89–91^.

Although the EMD- and IF-based techniques are more suitable than traditional methods for the analysis of non-linear and nonstationary data sets, they could easily be misused if their known limitations^21,92^, together with the assumptions they rely on, are not carefully considered. The endeavor of this study is to call attention to the main pitfalls encountered when implementing these techniques. Specifically, by examining a large number of studies pertaining to different fields, we have detected three critical factors that are often neglected or underestimated: boundary effects; presence of spikes/jumps in the original signal; signals generated by processes containing a high degree of stochasticity.

This paper is structured as follows: in “Problems with the boundaries” and “Spike pulses and jumps in the signal” we discuss the issues related to the boundary effects and to the presence of spikes/jumps, respectively. For each issue, we critically analyze a study that, in our opinion, represents a clear example of how either boundary conditions or spike/jumps should *not* be handled. In “Stochastic signals decomposition” we address the problem of the suitability of the EMD- and IF-based methods for the multiscale analysis of a stochastic signal.

In all the subsequent sections we present numerical examples, comparing the performance of the EMD, EEMD and IF algorithms. It is important to remind that, although in many instances the original EMD method produces results that are similar to those derived by its more evolved variants, we strongly discourage its usage. As we recalled previously, this method is particularly sensitive to noise and mode mixing. We suggest to implement either enhanced versions of the EMD technique^10,12–16^, or alternative methods such as the IF-based algorithms^17,18,23,25,27,29^.

## Problems with the boundaries

Like any signal processing technique, boundary conditions must be carefully addressed when implementing EMD and IF algorithms and their variants. This is equivalent to make assumptions about right and left extension of the signal, i.e. to extrapolate the time series beyond its boundaries. If not properly handled, end effects could arise, which result in anomalously high amplitudes of the IMFs and artifact wave peaks towards the boundaries.

We remark that identifying these errors is not always straightforward. This is because the IMFs are produced by subsequent subtraction from the original signal. Therefore, their sum always equals the original signal.

This problem was already pointed out by Huang and collaborators in their seminal work on the EMD^7^, and many approaches have been published since then to address it. Huang himself proposed the characteristic wave method^93^ and the extremum continuation method^94^. Other authors proposed, among many ones, the slope method^95^, the extremum image continuation method^96^, the artificial neural network method^97^, mirror extension coupled with support vector machine method^98^, and the extremum sequence extension^99^.

It is important to stress that is particularly difficult to estimate a priori the error contributions coming from the boundaries when implementing the EMD and derived methods, since a mathematical analysis of these techniques is still missing. To make things worst, there exist many alternative versions of these algorithms, each of which has its own peculiar way of handling boundaries. We mention here, for instance, the version by Yung-Hung Wang and collaborators (Fast EMD/EEMD Code https://in.ncu.edu.tw/~ncu34951/FEEMD.rar Research Center for Adaptive Data Analysis (RCADA), National Central University, Taiwan), the Patrick Flandrin and collaborators version (Matlab/C codes for EMD and EEMD http://perso.ens-lyon.fr/patrick.flandrin/emd.html Laboratoire de Physique, Ecole Normale Supérieure de Lyon, France), or the Mathworks Matlab official code (https://it.mathworks.com/help/signal/ref/emd.html). So far, there is no consensus on which version should be adopted to properly handle boundaries.

Regarding the IF-based methods, conversely, it is possible to a priori estimate the errors introduced by a specific boundary extension and to evaluate how they affect each IMF, since a complete and deep mathematical analysis of IF-based methods has been recently presented^18^. In particular, it is now possible to choose an optimal extension based on the specific features of the signal under study when we deal with the IF-based decompositions, reducing de facto the end effect errors in the decomposition. This is what it has been been done in^21^ for the periodical, symmetric (reflective), and anti-symmetric (anti-reflective) boundary extensions.

For the EMD-based methods, since a mathematically rigorous analysis of these methods is still missing, it is not possible to prove any general optimality of a given extension technique with respect to the others. Only a case by case analysis can be conducted at this stage. However, we can use the results derived from the rigorous mathematical analysis on the optimal pre-extension of a given signal for the IF-based methods, to extend the same signal also for the EMD-based algorithms.

Nevertheless, after pre-extension, the newly obtained data set $s_{\text {ext}}$ will be still finite. Therefore, some end effects may be present in the decomposition yet. One possibility is to force the extended signal to become periodical at the newly generated boundaries. In fact both IF and FIF are designed to decompose properly periodical signals without introducing any numerical error. Hence, we propose the following technique (*Signal Extension Algorithm* code available at www.cicone.com):

*Signal extension algorithm*

1. Subtract from the signal *s* its mean value *m*
2. Extend $s-m$ outside the boundaries in the preferred or optimal way, producing an extended signal $s_{\text {ext}}$ which is $\nu$ times longer than the original one
3. Multiply $s_{\text {ext}}$ by a characteristic function $\chi$ which has value one in the interval corresponding to the original signal *s* and goes smoothly to zero as we approach the new boundaries of $s_{\text {ext}}$
4. Add back the mean value *m* of the original signal
  $$\begin{aligned} s_{\text {new}}=\chi \cdot s_{\text {ext}} + m \end{aligned}$$

The produced signal $s_{\text {new}}$ is now periodical at the boundaries.

This approach allows to reduce the boundary errors in the IF-based algorithms, as known from the theory^21^, and in the EMD-based methods and shown by the following numerical simulations on synthetic and real life signals.

We point out that the proposed approach is similar in nature to a time-domain windowing technique. The main difference is that now we first pre-extend the signal and then apply a time-domain window. This window is constructed specifically to preserve unaltered the values at the center of the extended signal, which correspond to the original data. The proposed Signal Extension Algorithm allows to preserve and make use of all the information contained in the original data set, meanwhile reducing boundary effects errors.

Regarding the signal extension, step 2 of the proposed extension algorithm, in a recent paper^21^, as we mentioned previously, three kinds of extensions have been studied and compared: periodical, symmetric (reflective), and anti-symmetric (anti-reflective). In particular, they show the dependence of the end effects on the phase of the signal at the boundary. The result can be summarized as follows. If the slope of the signal at the boundary is close to zero, it is better to extend in a symmetric way. Whereas, when the slope is maximal in absolute value, it is better to extend in an anti-symmetrical way.

However, there are infinitely many other possible ways of extending a signal outside its boundaries besides periodical, symmetric and anti-symmetric extensions. What is the actual best or optimal way to properly extend a signal for IF-based methods remains, to the best of our knowledge, an open question. In this work we consider only these three kind of extensions and we choose for each example the optimal one among these three as suggested in^21^. The identification of the actual global optimal extension for each given signal for the IF-based methods and a detailed comparison with extension approaches proposed in the literature for the EMD-based techniques is out of the scope of this paper and we plan to tackle it in a future work.

Finally, regarding the choice of $\nu$, the number of times that we replicate the signal, its choice is a compromise between the need to reduce the border effects, and that of limiting the total length of the signal, keeping the computing time reasonable. In^21^ it was shown that border effects decrease exponentially with the distance from the edges of the signal. From this observation it follows that it sufficient to extend the signal with a $\nu$ from 0.5 to 5 of the original signal. The choice of the actual $\nu$ depends on the length of the original signal, the longer the signal the smaller we choose $\nu$, and on how low are the frequency we want to preserve, the lower are the frequency of interest the longer we extend the signal.

### Synthetic example

**Figure 1:**
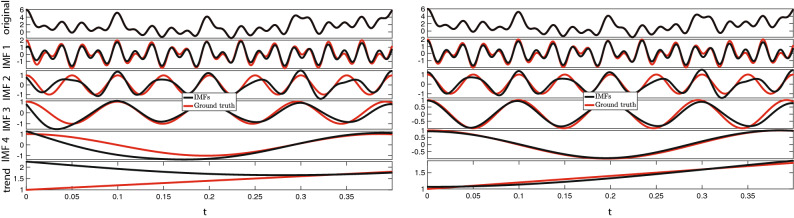
Boundary problems Synthetic Example. EMD results are compared with the analytical solution (“ground truth”). Left: EMD decomposition of the original signal (we show the first 1000 points). There are clear errors induced by EMD nearby the boundary. Right: EMD decomposition after pre-extending symmetrically left and right and made periodical the original signal, as described in “Problems with the boundaries”.

We consider the signal plotted in the top row of Fig. 1 which is given by

1 $$\begin{aligned} s(t)=2t+\cos (10t^2+100t)+\cos (60t)+\cos (40t)+\cos (t^2+20t)+\cos (0.5t^2+5t)+1\qquad t\in [0,\ 2\pi ] \end{aligned}$$

We first run the EMD algorithm included in matlab distribution 2018a and later versions, and produce the decomposition shown in the left panel of Fig. 1. To better visualize the end effects, we plot the first 1000 points only. Similar behaviors are present on the left boundary.

Issues are clearly visible nearby the boundary and they become more severe as the IMF frequency decreases. In order to reduce these end effects, we pre-extend the original signal. We make it five times longer than the original one, following the procedure presented above, since in this case the original signal is short enough and the computational complexity does not increase significantly in this way. We point out that in this case, since the signal has zero slope both on the right and left boundary, we opt to extend it symmetrically.

We feed this pre-extended signal to the EMD method to produce the decomposition shown in the right panel of Fig. 1. The end effects are visibly reduced.

Similar results are obtained using EEMD or FIF on this signal. The interested reader can find more details in the online supplementary material^100^.

In Table 1, we report the total computational time for the two EMD decompositions and the 2-norm of the relative differences between the ground truth and each IMF as well as the trend. This 2-norm quantifies the misfit between the ground truth and the IMF components produced via EMD.

**Table 1:** 2-norm of the relative differences between the ground truth and each IMF and the trend computed via EMD. Last row: total computational time.

2-norm rel. diff.	Original signal	Pre-extended signal
${\text {IMF}}_1$	0.3000	0.2725
${\text {IMF}}_2$	0.5107	0.4418
${\text {IMF}}_3$	0.5548	0.2634
${\text {IMF}}_4$	0.6569	0.0730
Trend	0.4007	0.0372
Computational time	0.0144 s	0.0247 s

This synthetic example shows that end effects mainly consist of artifact wave peaks at the onset (or at the end) of the IMFs: the higher the IMF index (i.e. the lower its frequency), the longer the wavelength of the spurious signal. It follows that such artifact can be recognized as a fictitious wave “propagating” towards the middle of the IMF, as one considers higher IMFs indexes. Additionally, end effects may also result into IMFs amplitude being larger than the original signal or even, in some cases, in the appearance of new IMFs containing frequencies not at all present in the original signal.

### Real life example

There is a huge amount of papers published in a wide variety of fields of research in which EMD-like methods are used to decompose signals. In some instances, we have identified a clear role of end effects in the derived decomposition^59,101–104^.

In the following, we examine, as an example, the results presented by Sarlis et al.^103^ where the authors themselves notice that some end effects are showing up in the decomposition. In that work, the analysed signal is the magnitude time series of the global seismicity for events of magnitude $M \ge 5.0$ .

**Figure 2:**
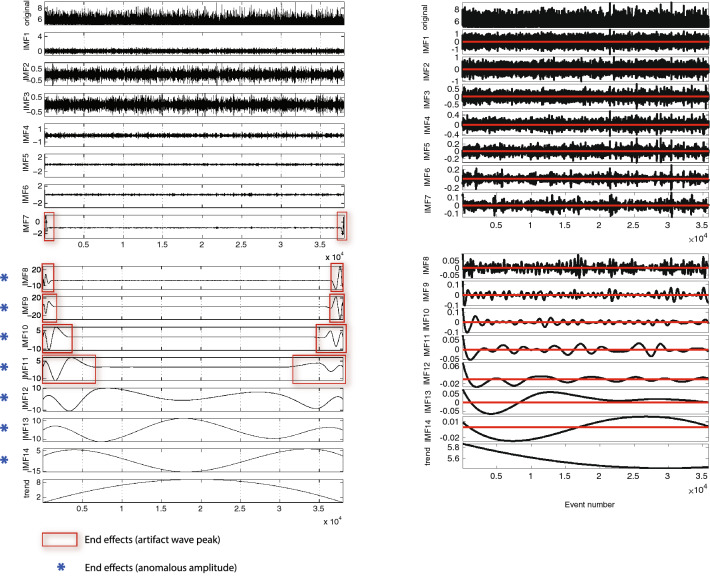
Boundary problems Real Life Example. Left: Adapted from Sarlis et al. paper^103^. EMD decomposition of the magnitude time-series of GCMT collected from 1 January 1976 to 1 October 2014. The red boxes highlight artifact wave peaks at the boundaries of the IMFs, while the blue asterisks pinpoint anomalous IMFs amplitudes (larger than the original signal). Right: Decomposition of the GCMT magnitude time-series from January 1st 1976 to October 1st 2014 produced using the EEMD function released on March 04 2009 by Zhaohua Wu. The red line in each panel represents the zero reference line. Total computational time: 213.9069 s.

In the left panel of Fig. 2, adapted from Fig. 1 in the original article, we use red boxes to pinpoint potential boundary effects, i.e. artifact wave peaks and anomalous amplitudes. Specifically, from IMF seven on, we notice the appearance of oscillations nearby the boundaries that have amplitudes different from the rest of the IMF and, more importantly, from the original signal. As an example, both the twelfth and thirteenth IMFs have values clearly oscillating in the interval $[-10,\ 10]$, whereas the original signal has values varying in the interval $[4.3,\ 9.08]$.

In order to reproduce their findings, we downloaded the catalog of global earthquakes ($M\ge 5.0$) from the Global Centroid Moment Tensor (CMT) Project (https://www.globalcmt.org/)^105,106^, for the period January 1, 1976–October 1, 2014. The global earthquake magnitude time series is shown in the top row of Fig. 2. We run the decomposition of this signal using both the EMD algorithm, included in matlab distribution 2018a and later versions, and the eemd algorithm (it can be downloaded from the official website of the Taiwanese Research Center for Adaptive Data Analysis https://in.ncu.edu.tw/~ncu34951/research1.htm and is contained in the repository https://in.ncu.edu.tw/~ncu34951/Matlab_runcode.zip) written by Zhaohua Wu in 2009^10^. This time we opt to set the number of elements in the ensemble to be 100 to speed up the calculations. The standard deviation is set, as suggested by the authors of the technique^10^, to 0.2. The outcome of these decompositions are pretty similar. We report in the right panel of Fig. 2 the one obtained via EEMD. The interested reader can find the EMD and FIF decomposition in the online supplementary material^100^.

In this case, no pre-extension of the signal was performed here, because the signal is highly erratic, and hence *pseudo-periodic*, as far as the border effects are concerned.

It is evident from the right panel of Fig. 2 that this decomposition do not contain the end effects obtained by Sarlis et al. (left panel of Fig. 2). The possible explanation is that they used some implementation of the EMD method which handles the boundaries in a way that induces the observed oscillations.

We point out that the authors of the original work also provided in their supplementary material^103^ the decomposition obtained using the EEMD code released by the Taiwanese Research Center for Adaptive Data Analysis. The decomposition they produced is compatible with the one shown in the right panel of Fig. 2.

## Spike pulses and jumps in the signal

If jumps or spikes are present in the time series, they can substantially affect the signal decomposition. As a matter of fact, when the EMD- and IF-based techniques are applied to a signal containing an impulsive change, their decompositions introduce oscillations at any frequency (ref. “Synthetic example” and “Real life example”). This is, mathematically speaking, expected and meaningful because any jump and spike can be represented locally as the summation of infinitely many frequencies. However, this way of representing a jump or spike is not necessarily meaningful from a physical point of view. Specifically, we warn that a physical interpretation of the derived decomposition could likely bring to conclusions apparently in contradiction with the causality principle.

### Caveats regarding the causality principle in analysing IMFs

A slippery problem is represented by the application of decomposition techniques, such as the EMD- and IF-based algorithms, to the identification of possible precursors of abrupt high-magnitude events like spikes and jumps. In many fields, as seismology, medical science, space weather or meteorology, guessing in advance when “catastrophic events” occur is very desirable. However, we warn the reader that using EMD- and IF-based decomposition techniques may be very misleading.

**Figure 3:**
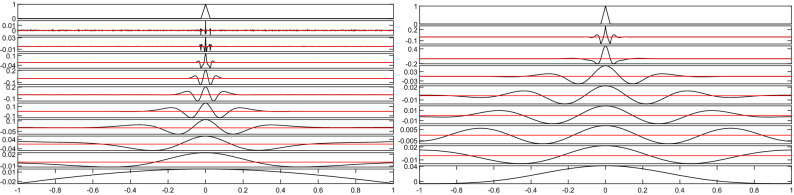
Spikes and jumps synthetic example. EEMD and FIF decompositions depicted in the left and right panels, respectively. The red line in each panel represents the zero reference line. Total computational time: 70.6450 s (EEMD), 0.1355 s (FIF).

The key point is that every abrupt change in amplitude, that necessarily is concentrated around some instant $t_{0}$ and takes place at small scales, can also be represented as the superposition of many components of different scales. In order to visualize this, just imagine to Fourier-decompose a peak as Dirac’s $\delta \left( t-t_{0}\right)$: a namely infinite assortment of frequencies will be necessary, hence involving also very large period components. The same thing happens for locally-characterized decompositions, like the ones produced using EMD- and IF-based methods, as shown in Fig. 3 of the following synthetic example: any peak around $t_{0}$ of a given original signal $x\left( t\right)$, appearing as a narrow bump in the original time series, will “broaden” on larger and larger intervals around $t_{0}$ when larger scale components are considered. In particular, if at some given “small” time scale $\ell$ the signal component $x_{\ell }\left( t\right)$ peaks within some interval $\left( t_{0}-\epsilon ,t_{0}+\eta \right)$, at some larger scale $\ell '>\ell$ the increment of the component $x_{\ell '}\left( t\right)$ will take place all along the interval $\left( t_{0}-\epsilon ',t_{0}+\eta '\right)$, with $\epsilon '\ge \epsilon$ and $\eta '\ge \eta$. All of this might induce the not-enough-careful scientist to imagine that, observing the large scale component $x_{\ell '}\left( t\right)$, a peaky behaviour can be already guessed to appear soon at time $t_{0}-\epsilon '\le t_{0}-\epsilon <t_{0}$, namely anticipating what the original signal $x\left( t\right)$, peaking at time $t_{0}$, will behave like. This is *clearly a mistake*: it would be as understanding that the $\delta$-like pulse force bouncing back a rubber ball from a wall is “sensed” at some distance from the solid wall by observing the time series of the force $F\left( t\right) =F\delta \left( t-t_{0}\right)$ exerted on the flying ball, being $t_{0}$ the precise time at which the collision takes place. This is blatantly false, as the force cannot be sensed in any way *before* the collision takes place. One can argue that, still, the “signal” $F\left( t\right) =F\delta \left( t-t_{0}\right)$ is indeed *composed by all* the large scale IMF addenda “anticipating” the impact, which is *indeed true*. The logical way out of this conundrum is the following: the time series of the $\delta$-like peak can be mathematically represented as the summation of components which apparently extend their influence further back in time as the scale size increases. However, from a physical stand point, this second interpretation makes no sense. A travelling ball cannot sense the presence of a wall in advance in any way. In particular, the large scale components produced in the mathematical decomposition of the force *F*(*t*) applied on the flying ball once it touches the wall, cannot be used as precursor of the collision.

**Figure 4:**
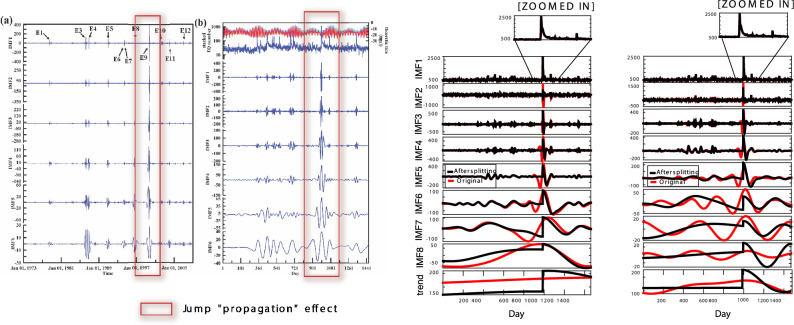
Spikes and jumps real life example. Adapted from Chen et al.^110^. Left: From top to bottom, the first six IMFs of the non-stacked data of the number of daily earthquakes from the Taiwanese catalog for events of magnitude $M_{L} \ge 3.0$ from 1978 to 2008. Center Left: The top row shows the temporal variations in theoretical Earth tides at the center of Taiwan with a period of 1462 days (red) and the stacked data of daily earthquake numbers (blue). The second to seventh row display the first six IMFs that were extracted from the stacked time series by Chen et al. In both panels, red boxes highlight the artifacts induced by a jump, in the original time series, which “propagates” through all the IMFs. Center Right and Right: Comparisons of the IMFs produced using EMD and FIF, respectively. The time signal in the first panel is zoomed in to highlight the jump. In red the decompositions obtained using the original stacked data set, and in black the ones obtained after splitting the time series into two disjoint subsets. In particular, we split it at the highest jump, in position 988. We use symmetric extension to produce all the results. Total computational time: 0.3479 s (EMD—splitted signal), 0.0511 s (EMD—original data set), 0.3243 s (FIF—splitted signal), 0.0526 s (FIF—original data set).

The examples reported in the following “Synthetic example” and “Real life example” all clearly suffer from this problem. It is actually incorrect to use information from the large scale components to infer the occurrence time of the peak, before it has occurred. This is because those low frequency components, necessary from a mathematical stand point to re-produce the spike or jump *a posteriori*, do not have the same aspect if the original signal $x\left( t\ll t_{0}\right)$ does not show any peak yet, as shown in Fig. 4.

This does not mean that multiscale decomposition of some time series $x\left( t\right)$, being it performed via EMD- or IF-like methods, or even the standard wavelet or Fourier transform, cannot be of use in investigating the physical properties of the process producing the time series. What multiscale decomposition and their study can be of use for is the detection of behaviour and statistics *within the time interval sampled*: so, as a time series represents what a probe encounters as the phenomenon evolves in a given time interval $\left[ t_{\mathrm {i}},t_{\mathrm {f}}\right]$, multiscale statistics of the time series reveals what has taken place at the different scales in the whole $\left[ t_{\mathrm {i}},t_{\mathrm {f}}\right]$: this may be of great use in understanding, e.g., whether, turbulence^47–49,107^, intermittency^46^ or critical behaviour^108^ *have taken place* in $\left[ t_{\mathrm {i}},t_{\mathrm {f}}\right]$. We will come back on this topic in “Stochastic signals decomposition”.

In the following we first show, by numerical simulation, how the presence of a spike may influence the decomposition (“Synthetic example”). Then, we examine one of the literature studies to stress the caveats about spikes and jumps proper handling in the decomposition of a signal (“Real life example”).

### Synthetic example

We start showing, by means of a simple numerical simulation, how the presence of even a single spike may influence the signal decomposition. Specifically, we simulate a constant-amplitude signal with a impulsive spike, shown in the top row of Fig. 3, and then we decompose it by means of both the EEMD and the FIF techniques. For the EEMD method we set the standard deviation to 0.2 and the dimension of the ensemble to 800, to reduce as much as possible the noise-related IMFs. Results are shown in Fig. 3.

We point out that this example allows to understand also how a jump, or multiple ones, can influence the decomposition of a signal. In fact, if we imagine covering with our hand the second half of Fig. 3 left or right panels, what we see is the beginning of a jump and its corresponding decomposition. In fact a spike can be viewed as two consecutive jumps, one going up and the other going down. From this observation it follows that there is no need to present here a separate example to show the influence of a jump in the decomposition of a signal. At the same time, it is important to underline that the idea of interpreting a spike as two consecutive jumps is not a practical way of dealing with spikes contained in a data set. The actual handling of spikes and jumps present in a data set differs widely, in fact, as explained in the following section.

One of the most important lesson that we can learn from this example is that the information contained in a single spike diffuses quickly far away in time/space from the location of the spike when we decompose it. Furthermore, from Fig. 3 we observe that, if we consider the peak values in each IMF component, the errors are distributed practically uniformly over all frequencies. The lower is the frequency the more far away from the spike location we have an impact in the decomposition. Researchers could be tempted to assume that such impact regards only low frequency IMF components. But there is not a single frequency which is not impacted in the decomposition by the presence of even a single spike, and the more the spikes are, the worse the situation becomes. So, regardless the context in which the signal is generated, it is always strongly advisable to decompose both the signal as it is and the signal after an appropriate pre-processing in order to understand and estimate the impact of spikes and jumps in the decomposition.

### Real life example

We have found several studies published in the literature where signals containing spikes or jumps are not carefully handled^38,109–112^.

The proper identification of spikes position in signals has been already studied in the literature, for instance in^113,114^, and for the jumps the so called essentially non-oscillatory (ENO) technique was developed in computational fluid dynamics to capture shock positions^25,115^, and it can be adopted in this context. If on the one hand some approaches have been proposed on how to remove spikes, on the other hand the question of how to properly handle jumps have never been raised in the context of EMD- and IF-based methods decompositions, to the best of the authors knowledge.

For this reason, in the following, we focus on the jump handling. In particular we examine, as an example, one of the decompositions presented by Chen et al.^110^, where the authors analyse the number of daily earthquakes time series ($M_{L} \ge 3.0$) occurred in Taiwan in the period 1978-2008, which present naturally jumps.

In Fig. 4 (left and center left panels, adapted from Fig. 3 in the original article) we use red boxes to pinpoint jump effects, i.e. artifact waves “propagating” throughout all the IMFs.

In the following, we consider the stacked data set analysed by the authors of the original work, which is shown in the top row of Fig. 4 center left panel. Here, the highest jumps correspond to the sequences of events triggered by the largest earthquakes.

We decompose this signal using both EMD (we used the EMD algorithm included in matlab distribution 2018a and later versions) and FIF (available at www.cicone.com) in two ways: without any “pre-processing”, as in the original article^110^, and with a “pre-processing”, which consists in splitting the time series in “before” and “after” the jump, and symmetrically extending the two disjoint subsets. Regarding the jumps and spikes identification and their localization, as we mentioned previously, many works have been published in the literature where different techniques have been proposed, e.g.^113,114^. Therefore in this work we assume that their localization is known.

The outcome of these decompositions are shown in Fig. 4, center right and right panels. Here we plot in red the decompositions produced using the original stack data set, and in black the decomposition after splitting and symmetrically extending the two disjoint subsets.

It is evident from these results that an improper handling of the jump can severely affect the returned decomposition at time $t<t_0$. Specifically, the lower the IMF frequency, the further the jump has influence back in time.

Based on these evidences, we suggest to always compare the decomposition obtained before and after “pre-processing” the signal. Regarding the pre-processing, if a jump is present we propose to split the signal in two subsets, before and after the jump. Whereas, if one or more spikes are present, they can be localized and removed following what has been already suggested in the literature, e.g.^113,114^.

We also observe that the presence of the main jumps and spikes in a signal can be detected looking at the IMF components of the original signal in time domain all together, ref. Figs. 3 and 4. However this approach is not enough robust to help in the identification of secondary spikes and jumps which can affect badly the signal decomposition and mask other nonstationarities. So it is always advisable to use an ad hoc method for the spikes and jumps identification and removal, and to compare the decomposition before and after pre-processing.

## Stochastic signals decomposition

When dealing with real data, the first question we should ask ourselves is: can we apply these decomposition techniques to the signal under analysis?

The EMD- and IF-based methods proved to be well suited for the analysis of signals coming from diffusion processes like heat or wave equations and, more in general, systems whose behavior can be described by differential equations with oscillatory solutions. Whereas, a *stochastic signal* is missing enough regularity to be described by a mathematical model based on ordinary or partial differential equations. It is therefore an open problem to assess whether the techniques, on which this paper is focused, can successfully reproduce the features of the signal at different scales. In fact, while it is always possible to decompose a signal by EMD- and IF-based methods, a physical interpretation of the derived IMFs and their features has to be done with care, even in the case of deterministic signals; the stochastic signal case may appear even more uncertain.

As a first observation, one should point out that stochastic signals can be analysed sensibly with two purposes: either, *to separate from the signal a deterministic part of it*, that could be used to model part of the phenomenon via non-stochastic tools (e.g., systems of differential equations); or *to study statistical characteristics of the signal*. The application we want to discuss here pertains to this latter purpose.

Here the EMD and FIF techniques are applied to a synthetic stochastic time series largely used to mimic *turbulent signals*, both in fluid and plasma dynamics, namely the *p-model* (see, for instance:^116^ and^117^). Even if EMD and FIF decomposition is generally used to recognize and reconstruct the mathematical form of different components of a time series, so to understand the different contributions to a given phenomenon, this is not the aim here. Indeed, the p-model is constructed by summing functions that do not meet the characteristics of the components that can be reconstructed via EMD or via FIF. Still, decomposing the p-model signal via such techniques, in order to study its *statistical properties at diffrent scales*, allows to provide meaningful insights in the data sets, as shown below.

The p-model is a simple branching model: still, it is extremely powerful in mimicking the irregular and intermittent distribution of energy in turbulent media (^118,119^).

The p-model construction starts from the distribution

2 $$\begin{aligned} {\left\{ \begin{array}{ll} & u_{0}\left( x\right) =E\left[ \theta \left( L-x\right) -\theta \left( -x\right) \right] \\ & \int u_{0}\left( x\right) dx=E>0 \end{array}\right. } \end{aligned}$$

i.e., a piecewise constant distribution that is equal to *E* in the interval $I=\left[ 0,L\right]$ and zero outside. The $\theta$s are Heavyside step functions. Then, the interval *I* is divided into two subintervals $I_{11}=\left[ 0,\frac{L}{2}\right]$ and $I_{12}=\left[ \frac{L}{2},L\right]$, and the quantity *E*, contained in the whole original interval, is distributed “randomly” in $I_{11}$ and $I_{12}$, so that the overall amount remains the same. In order to do so, a parameter $p\in \left[ 0,1\right]$ is defined, and two weights $w_{11}$ and $w_{12}$ are chosen, whose value is randomly chosen between 2*p* and $2\left( 1-p\right)$, so that $\left( w_{11}+w_{12}\right) \frac{E}{2}=E$. In doing so we define the distribution

3 $$\begin{aligned} {\left\{ \begin{array}{ll} & u_{1}\left( x\right) =\frac{E}{2}\left[ w_{11}\chi _{11}\left( x\right) +w_{12}\chi _{12}\left( x\right) \right] \\ & \chi _{11}\left( x\right) =\theta \left( \frac{L}{2}-x\right) -\theta \left( -x\right) \\ & \chi _{12}\left( x\right) =\theta \left( L-x\right) -\theta \left( \frac{L}{2}-x\right) \end{array}\right. } \end{aligned}$$

The $u_{1}\left( x\right)$ distribution is zero outside $I=\left[ 0,L\right]$ and it is considered “the first generation” of the p-model. The branching process $u_{0}\mapsto u_{1}$ is repeated as $u_{1}\mapsto u_{2}$, by subdividing $I_{11}\overset{\mathrm {def}}{=}\left[ 0,\frac{L}{2}\right]$ and $I_{12}\overset{\mathrm {def}}{=}\left[ \frac{L}{2},L\right]$ into two halves each, and repeating the random assignment of the weights 2*p* and $2\left( 1-p\right)$ to each half of $I_{11}$ and of $I_{12}$. By recursively repeating the previous steps, at the *n*-th step the distribution $u_{n}\left( x\right)$ is derived. This is constant on each of the $2^{n}$ intervals $I_{n,i=1,2^{n}}$, each of which has length $\ell _{n}=\frac{L}{2^{n}}$, and has the same integral as all the other ones: $\int u_{n}\left( x\right) dx=E,\ \forall n$. For a thorough explanation of the branching process just sketched, with explicit calculation, the interested reader can refer to the paper by Materassi et al.^120^

The profiles $u_{n}\left( x\right)$ produced at a suitably high value of *n* by the p-model were proved to be useful to mimic the distribution of kinetic energy in fluid turbulence^118,119^, or that of magnetic energy in MHD or ionospheric turbulence^121–123^. Moreover, the choice of the parameter *p*, with which we defined the weights $w_{11}$ and $w_{12}$ in (3), tunes the *degree of intermittency* of the final result $u_{n}\left( x\right)$, as it regulates how uneven the partition of the amount *E* is from the $\left( n-1\right)$-th to the *n*-th generation: $p=\frac{1}{2}$ reproduces Kolmogorov’s non-intermittent K41 theory^119^, while $p\rightarrow 0$ and $p\rightarrow 1$ gives rise to more and more intermittent distributions, with a mirror symmetry between $p\in \left[ 0,\frac{1}{2}\right)$ and $p\in \left[ \frac{1}{2},1\right]$. Last, but not least, the p-model distribution shows a well known multi-fractal singularity spectrum^122,124,125^. All those reasons render the p-model series a suitable test bed for multiscale analysis techniques, as EMD and IF, because the ground truth is an intermittent signal of perfectly known characteristics, and the extent to which those are retrieved by the various analysis tools clearly emerges.

In the following, we consider a signal $p\left( x\right)$ obtained from the summation of $n=12$ generations $u_{k=0,...,12}$, *each representing a different realization* of a p-model $p\left( x\right) =\sum _{k=0}^{12}u_{k}\left( x\right)$. In doing so we produce a signal which contains several scales which are enough “orthogonal” (i.e. independent) to each other. The sum $p\left( x\right)$ clearly contains information about each of the *k*th generations corresponding to $n=12$ different p-models, from the 0th to the 12th one, because it is formed by these addenda.

**Figure 5:**
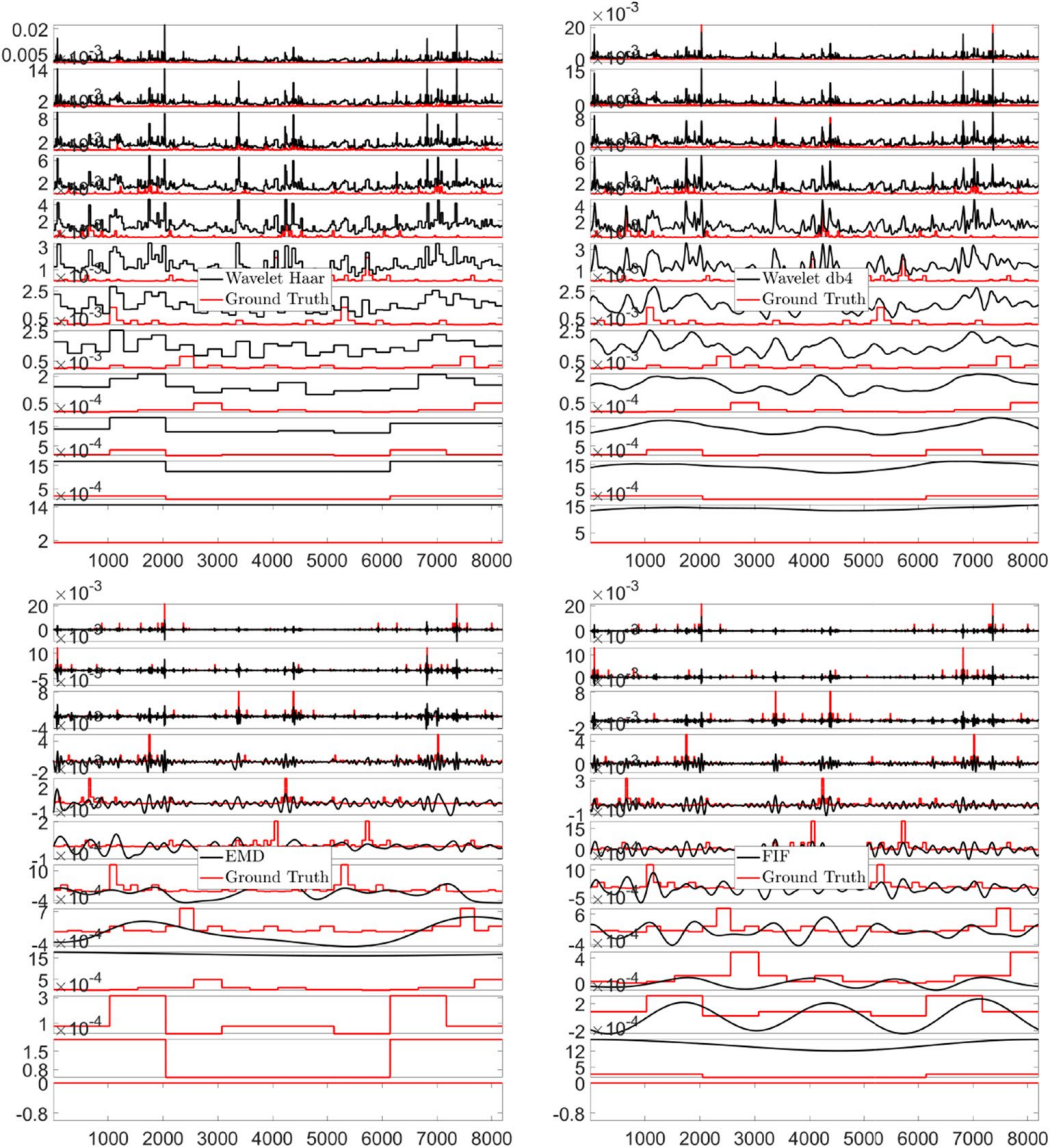
Stochastic signals—P-model Example. From Top left to Bottom right corner, Haar and db4 Wavelet, EMD and FIF decompositions, respectively. In solid red the corresponding ground truth $u_{k}$ generations. In each panel, the x-axis plots the sample points, and the y-axis the IMFs ordered from top to bottom.

As a next step, we decompose this signal via the DWT, using both “Haar” and “Daubechies 4” (db4) bases, EMD and FIF algorithms. In this work we compare the performance of the EMD- and IF-based methods with that of the discrete wavelet, since wavelets are a very well established tools in the study of turbulent signals^126^. Moreover, regarding the choice of the wavelet bases, the “Haar” wavelets are particularly suited to treat stepwise-function based signals, as the single components $u_k$ are; whereas the use of “Daubechies” decomposition is well established in fluctuation analysis^127^. We call $\left\{ \psi _{h}\right\}$ the set of functions along which the signal is decomposed, so that $p\left( x\right) =\sum _{h}c_{h}\psi _{h}\left( x\right)$. In our analysis, the functions $\psi _{h}$ are DWT generated levels, EMD or FIF IMF functions. The *h*th-scale filtered component of the true signal $p\left( x\right)$ is defined as $p_{h}(x)\overset{\mathrm {def}}{=}c_{h}\psi _{h}\left( x\right)$. We show these decompositions in Fig. 5. It is evident that none of the aforementioned techniques is able to extract components which resemble the corresponding ground truth $u_{k}$ generations, and on the other hand we already anticipated that retrieving those addenda *is not* the purpose of our analysis.

One may argue that, for real life data sets, probability distributions, such as the exponential one, represent the large-scale randomness which could arise from the superposition of nonrandom processes at smaller scales. While this could be correct in theory, the previous simulation shows that DWT, EMD- and IF-based algorithms might not help researchers in identifying the exact origin of a data set. These techniques will always produce a decomposition of the signal, no matter what the process behind it is.

Finally, we compare the ability of all these techniques in reconstructing *multiscale statistical features* of the given signal, compared to the ground truth ones. We study, in particular, the standard deviation $\sigma \left( p_{h}(x)\right)$, the skewness $S\left( p_{h}(x)\right)$ and the excess of kurtosis $K\left( p_{h}(x)\right)$, as a function of the scale *h*. Moreover, we include the total energy pertaining to the *h*-th scale $\mathscr {E}\left( p_{h}(x)\right)$, and the inner product between two nearby scales $\mathscr {C}\left( p_{h}(x)\right)$, which we calculate as:

$$\begin{aligned} {\left\{ \begin{array}{ll} & \mathscr {E}\left( p_{h}(x)\right) =\int \left| p_{h}(x)^{2}\left( x\right) \right| dx,\\ & \mathscr {C}\left( p_{h}(x)\right) =\int p_{h}(x)(x)p_{h-1}\left( x\right) dx \end{array}\right. } \end{aligned}$$

**Figure 6:**
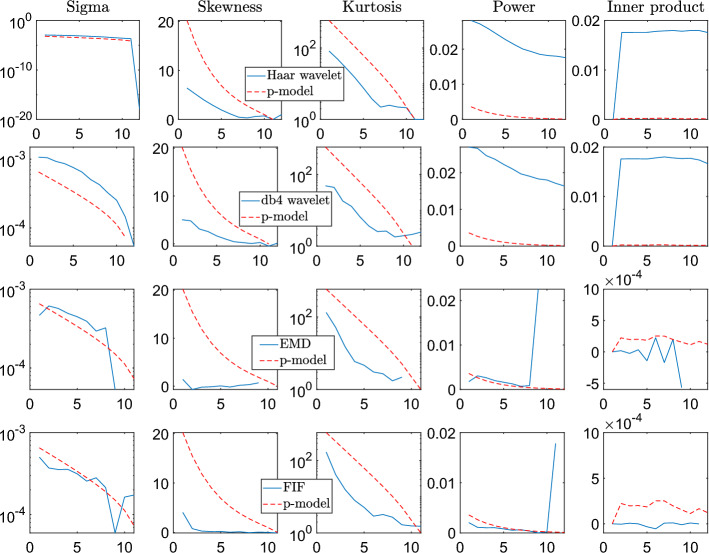
Stochastic signals—P-model Example. From Top to Bottom rows, multiscale statistical analysis of the Haar and Daubechies 4 Wavelet, EMD, and FIF decompositions, respectively. Different statistical quantities are plotted with respect to the IMF number, from left to right columns: standard deviation (sigma), skewness, kurtosis, power and inner product of two subsequent levels. In dashed red the ground truth values.

The derived multiscale statistics are presented in Fig. 6.

A *stochastic multi-scale (multifractal) signal* as the p-model, or any highly turbulent natural signal, can be conveniently characterized by the statistical properties it shows when it is zoomed in at different time or space scales. For instance, multi-scale analysis of the statistical properties of turbulent signals in geophysics and plasma science may unveil which dynamical processes are at work to produce what the instruments measure. The purpose here is to show what happens when the “zoom-in” tool are EMD- and IF-based techniques, and the result is that the multi-scale statistical properties recognized are in agreement with what found via other more traditional (and literature-consolidated) techniques. From Fig. 6 we see that the behavior, scale by scale, of signal variance, skewness and kurtosis, and the scalar product between adjacent scales, is caught rather well when we filter the signal with EMD and FIF methods. In particular these techniques prove to help in reconstructing the trend of $\sigma (p_h)$, $S(p_h)$ or $K(p_h)$ functions, which is the main objective in real life turbulence study.

We observe that the DWT has troubles regardless the basis we select in reproducing the exact multiscale statistical features values, but it provides the right trends. EMD and FIF decompositions, instead, prove to be more accurate in replicating the standard deviation and power of the different components. The skewness values, as expected, are close to zero. These methods, in fact, decompose signals into simple and pseudo-sinusoidal IMFs which are symmetric with respect to the horizontal axis. Furthermore, the inner products between subsequent IMFs tends to zero since the EMD and FIF methods produces components which tend to be almost orthogonal each other, as the ground truth levels which have been obtained by different p-models. Regarding the kurtosis, EMD and FIF, as well as DWT, are all able to properly reproduce its trend as a function of the scale. We therefore conclude that *all* the multiscale analysis tools compared in this example are able to detect the *intermittency of the signal* under analysis^119^.

Furthermore, the previous example shows that either DWT, or EMD or FIF techniques leave their characteristic signatures in the multiscale analysis of a given signal. Like, for instance, in the case of the skewness values for the IMFs produced using EMD- and IF-based methods. We explain, in fact, that these techniques are designed to produce simple components which have symmetric envelopes with respect to the horizontal axis. Nevertheless, both EMD and FIF provide a meaningful insight in the multiscale statistical analysis and trends of the signal under study, even in presence of strong stochasticity.

Physical signals may not only be *stochastic*, but also result out of the superposition of a deterministic part with noise. In general, as far as “noise” is expected to be a high frequency component, EMD and FIF techniques should separate it from low frequency deterministic parts, and then treated as here described. The case of superposition of noise and high frequency deterministic parts should be treated in more specific ways, see for instance^128^.

This example should have clarified the limits as well as the potentialities of these techniques when analysing signals characterized by some degree of stochasticity. As already reported, in real-life applications particular care must be taken when interpreting IMFs in physical terms. Based on the results proposed in this section, we discourage to blindly decompose and analyse a signal when its degree of stochasticity is not well known.

## Conclusions

The EMD- and IF-based methods proved to be more suitable than traditional methods for the analysis of nonlinear and nonstationary signals. Their relevance is witnessed by the large number of studies employing them. However, like any other technique, these methods rely on assumptions and have limitations that, if neglected, can severely affect any interpretation based on the returned decomposition. In this work, we examine the main pitfalls and provide caveats for the proper use of the EMD- and IF-based algorithms. Specifically, we address the problems related to boundary errors, to spikes and jumps in the signal and to the analysis of highly-stochastic data sets.

- Boundary conditions may influence the decomposition of a signal to an extent that increases with the component scale. If not properly handled, they could lead to an artefact-prone decomposition of the original signal. This problem has been studied rigorously in the literature^21^ for the IF-based methods, but not for the EMD-based algorithms. However, based on the results obtained so far for the IF-based methods, we can reduce the impact of the possible boundary errors for both IF- and EMD-based algorithms by properly pre-extending the signal under study. For this reason, here we propose a new approach for the pre-extension of a given signal. This method is based on the assumption that the optimal way to extend the signal at the boundaries is known. So far, the only extensions studied rigorously in the literature are the periodical, symmetrical and anti-symmetrical ones for the IF-based methods. How to optimally extend in general a signal outside its boundaries is still an open problem that we leave to a future research project.
- Spikes, including outliers and jumps, can have a big impact on the decomposition, as shown in the examples presented in this work. We show that they could lead, as for the boundary conditions, to an artefact-prone decomposition of the original signal. Moreover, we encourage researchers to be extremely careful when conducting any precursory analysis based on signal decomposition techniques, such as EMD and IF methods. When dealing with a signal containing spikes, the optimal solution would be to study its decomposition before and after removing the spikes from the signal itself. Whereas, when a jump is present in the data set, a good practice would be to split it into *before* and *after* the jump, analyse the two portions separately, and compare the outcome of this decomposition with the one of the original signal.
- In this work, we raise the question of whether the EMD- and IF-based methods are suitable for the analysis of highly stochastic signals. Although the derived decomposition is always correct from a mathematical stand point, it may be the case that there is not a corresponding evident physical meaning of each IMF.
  As a matter of fact, when the signal is originated by processes whose behavior can be described by differential equations with oscillatory solutions, EMD- and IF-based techniques produce a decomposition which is meaningful from both a mathematical and a physical standpoint. Whereas, when the process underlying the signal that we want to analyse is characterized by a high degree of stochasticity, the ability of these techniques in separating properly the different scales becomes less clear. In this paper we consider, as an example, the multiscale statistical analysis of the decompositions produced by the DWT-, EMD- and IF-based methods of a stochastic signal obtained as solution of a p-model. This model has been proposed and used in the literature to generate signals which mimic the behavior of irregular and intermittent distribution of energy in turbulent media. The EMD- and IF-based methods proved to have good performance from a multiscale statistical analysis prospective. It remains, however, an open problem to understand up to which degree of stochasticity these techniques are able to reproduce with a good accuracy the single components contained in a given signal. We plan to study this matter in a forthcoming work.

From all these results it is evident that it can be risky to blindly run the decomposition of a nonstationary signals by means of the EMD- and IF-based techniques and using the results without carefully considering the aforementioned limitations. However, the right handling of these techniques allows the users to fully exploit their potentialities in the analysis of nonstationary signals.

## Supplementary information

Supplementary Information.
