# Supplementary material for: New insights and best practices for the successful use of Empirical Mode Decomposition, Iterative Filtering and derived algorithms
Source: Sci Rep. 2020 Sep 16;10:15161. doi: 10.1038/s41598-020-72193-2 (PMC7495475; doi:10.1038/s41598-020-72193-2)
Supplement: Supplementary file 1 — Supplementary Information. [file 41598_2020_72193_MOESM1_ESM.pdf]

Supplementary Material: New insights and best practices for the successful use of Empirical Mode Decomposition, Iterative Filtering and derived algorithms

Angela Stallone[1], **Antonio Cicone**[2,*], **and Massimo Materassi**[3]

[1]Istituto Nazionale di Geofisica e Vulcanologia (INGV), Roma, 00143, ITALY
[2]Istituto di Astrofisica e Planetologia Spaziali dell'Istituto Nazionale di Astrofisica (IAPS-INAF), Via Fosso del Cavaliere 100, 00133, Roma, ITALY
[3]Istituto dei Sistemi Complessi del Consiglio Nazionale delle Ricerche (ISC-CNR), via Madonna del Piano 10, 50019, Firenze
[*]antonio.cicone@inaf.it

ABSTRACT

In this supplementary document we provide: EEMD and FIF decomposition of the synthetic signal introduced in Section 2.1; EMD and FIF decomposition of the GCMT time series analyzed in[1].

Boundary Conditions Synthetic Example

We decompose the signal given in Section 2.1 using the EEMD code written by Zhaohua Wu in 2009[2], which can be downloaded from the official website of the Taiwanese Research Center for Adaptive Data Analysis https://in.ncu.edu.tw/~ncu34951/research1.htm (the EEMD code we tested is contained in the repository https://in.ncu.edu.tw/~ncu34951/Matlab_runcode.zip). In the first test, we set the number of elements in the ensemble to 800 and the standard deviation to 0.2, as suggested in[2]. The returned decomposition is shown in the left panel of Figure S1. Issues at the boundaries are clearly evident. In the second test, we pre–extend the signal symmetrically up to five times the length of the original signal and we make it periodical, as described in Section 2. The returned decomposition is shown in the right panel of Figure S1. The end effects are now clearly reduced.

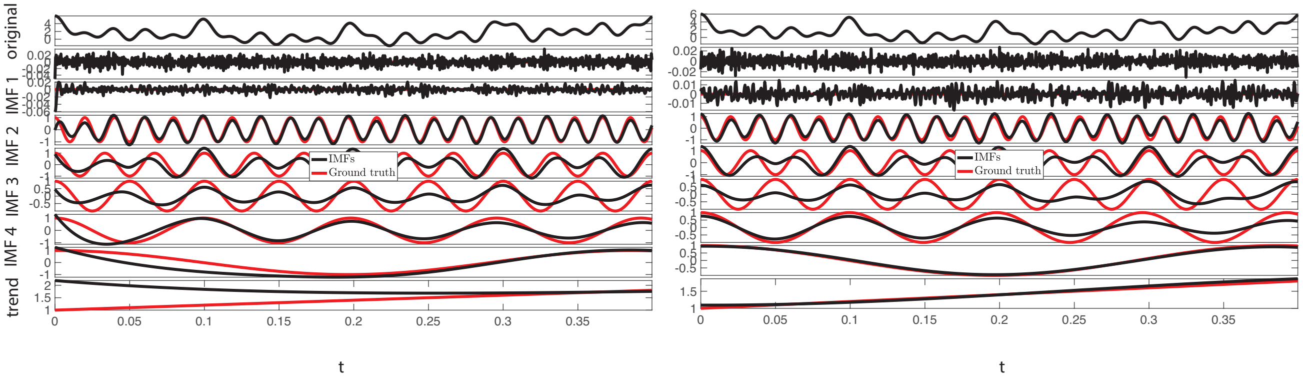

Figure S1. Boundary Conditions Synthetic Example. Left: EEMD decomposition of the original signal. We observe errors nearby the boundaries, together with mode–splitting. The first three IMFs are clearly affected by the perturbations at the edges. Furthermore, oscillations in IMFs 9–11 are probably artefacts induced by the boundary errors propagating across the whole time range, and they are affected by the mode–splitting issue. Right: EEMD decomposition when the signal is pre–extended symmetrically. For simplicity, we show only the first 1000 points of the original signal. The first three IMFs are induced by the perturbations at the edges. The trend is split into 6 IMFs

In both the examples shown in Figure S1, we observe the typical issues related to the use of EEMD algorithm: mode splitting and noise–related IMFs.

In Table 1, we report the total computational time for the two EEMD decompositions and the 2-norm of the relative differences between the ground truth and each IMF as well as the trend. This 2-norm quantifies the misfit between ground truth and IMF components produced via EEMD.

2–norm rel. diff.	Original signal	Pre-extended signal
IMF_1	0.3534	0.2664
IMF_2	0.2269	0.1638
IMF_3	0.2726	0.3343
IMF_4	0.6275	0.7322
IMF_5	0.6832	0.8136
IMF_6	0.4605	0.4407
IMF_7	0.5840	0.0680
Trend	0.3662	0.0309
Computational time	96.4756 s	343.4722 s

Table 1. 2-norm of the relative differences between the ground truth and each IMF and the trend computed via EEMD. Last row: Total computational time.

We now decompose the signal by means of the FIF algorithm (available at `www.cicone.com`). Results are shown in the left panel of Figure S2. As explained in Section 1, FIF algorithm automatically enforces a periodical extension at the edges. The derived IMFs are affected by errors at the boundaries, to an extent which is greater for the lower frequency components. This is clearly due to the fact that the original signal is not periodical at the boundaries. If, however, we symmetrically pre–extend the signal as in the previous example, the errors at the boundaries are drastically reduced, as shown in the right panel of Figure S2.

In Table 2, we report the total computational time for the two FIF decompositions and the 2-norm of the relative differences between the ground truth and each IMF and the trend.

2–norm rel. diff.	Original signal	Pre-extended signal
IMF_1	0.2386	0.0498
IMF_2	0.1811	0.1148
IMF_3	0.2340	0.1052
IMF_4	0.2583	0.0216
IMF_5	0.8545	0.0696
Trend	0.4881	0.0369
Computational time	0.3498 s	1.4687 s

Table 2. 2-norm of the relative differences between the ground truth and each IMF and the trend computed using FIF. Last row: Total computational time.

We stress that FIF algorithm does not have issues related to noise–related IMFs, for it does not require to perturb the original signal, like the EEMD algorithm. This characteristic makes the FIF method extremely faster than both EEMD and EMD methods[3].

We remark that, regardless the method implemented to decompose the signal, it is advisable to pre–extend it symmetrically and to make it periodical, as explained in Section 2.

Boundary Conditions Real Life Example

Here we implement the EMD and FIF algorithms to decompose the signal analyzed in[1]. This consists of the global earthquake magnitude ($M \geq 5.0$) time-series from the Global Centroid Moment Tensor (CMT) Project (`https://www.globalcmt.org/`)[4,5], for the period January 1, 1976 – October 1, 2014. The decomposition returned by the EMD algorithm (included in MATLAB distribution 2018a and later versions) is shown in Figure S3, left panel.

The decomposition returned by the FIF algorithm is show in Figure S3, right panel. In order to reduce the boundary effects, we first extend the signal at the boundaries and then we make it periodical. In this example, given the apparent absence of low frequency oscillations, we decide to extend it up to three times the length of the original signal. Furthermore, the symmetric-type extension proves to be the best option in this case[6].

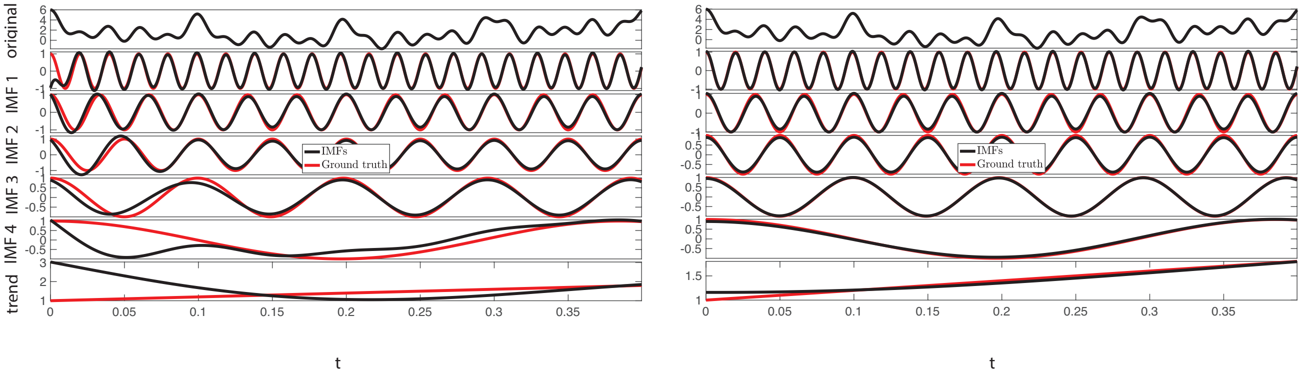

Figure S2. Boundary Conditions Synthetic Example. Left: FIF decomposition of the original signal. By construction, FIF algorithm extends the signal periodically outside the boundaries. Right: FIF decomposition when the signal is pre–extended symmetrically. For simplicity, we plot the first 1000 points of the original signal.

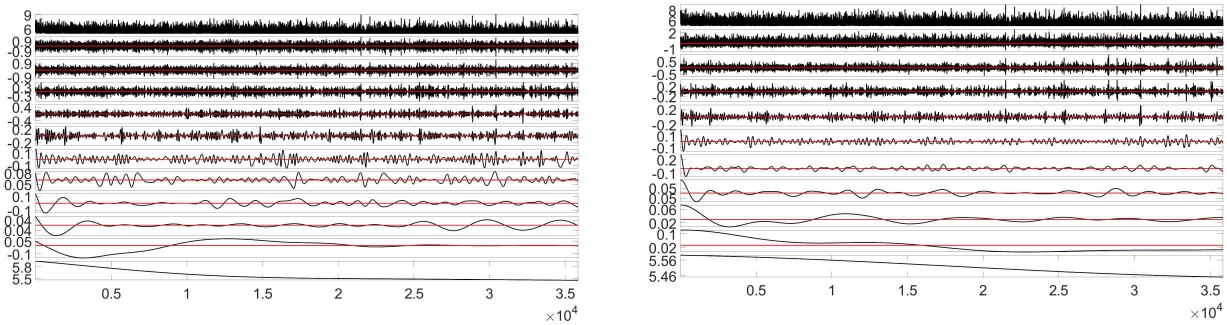

Figure S3. Boundary problems Real Life Example. EMD, left, and FIF decomposition, right, of the global EQ magnitude ($M \geq 5.0$) time-series from the Global Centroid Moment Tensor (CMT) Project. Total computational times: 0.2116 s (EMD), 0.6391 s (FIF).

References

1. Sarlis, N. V., Skordas, E. S., Mintzelas, A. & Papadopoulou, K. A. Micro-scale, mid-scale, and macro-scale in global seismicity identified by empirical mode decomposition and their multifractal characteristics. *Sci. reports* **8**, 9206, DOI: https://doi.org/10.1038/s41598-018-27567-y (2018).
2. Wu, Z. & Huang, N. E. Ensemble empirical mode decomposition: a noise-assisted data analysis method. *Adv. adaptive data analysis* **1**, 1–41, DOI: https://doi.org/10.1142/S1793536909000047 (2009).
3. Cicone, A. Iterative filtering as a direct method for the decomposition of nonstationary signals. *Numer. Algorithms* DOI: https://doi.org/10.1007/s11075-019-00838-z (2020).
4. Dziewonski, A. M., Chou, T. A. & Woodhouse, J. H. Determination of earthquake source parameters from waveform data for studies of global and regional seismicity. *J. Geophys. Res. Solid Earth* **86**, 2825–2852, DOI: https://doi.org/10.1029/JB086iB04p02825 (1981).
5. Ekström, G., Nettles, M. & Dziewoński, A. M. The global cmt project 2004–2010: Centroid-moment tensors for 13,017 earthquakes. *Phys. Earth Planet. Interiors* **200**, 1–9, DOI: https://doi.org/10.1016/j.pepi.2012.04.002 (2012).
6. Cicone, A. & Dell'Acqua, P. Study of boundary conditions in the iterative filtering method for the decomposition of nonstationary signals. *J. Comput. Appl. Math.* **373**, 112248, DOI: https://doi.org/10.1016/j.cam.2019.04.028 (2020).
